# Maxillary Growth Encircling the Central Incisor Crown

**DOI:** 10.5005/jp-journals-10005-1065

**Published:** 2010-08-17

**Authors:** K Anbarasi, S Sathasivasubramanian, N Malathy, N Nandakumar

**Affiliations:** 1 Senior Lecturer, Department of Oral Medicine and Radiology, Sri Ramachandra Dental College, Sri Ramachandra University Porur, Chennai, Tamil Nadu, India; 2 Professor and Head, Department of Oral Medicine and Radiology, Sri Ramachandra Dental College, Sri Ramachandra University, Porur, Chennai, Tamil Nadu, India; 3 Professor and Head, Department of Oral Pathology, Sri Ramachandra Dental College, Sri Ramachandra University, Porur Chennai, Tamil Nadu, India; 4 Associate Professor, Department of Oral and Maxillofacial Surgery, Sri Ramachandra Dental College, Sri Ramachandra University, Porur, Chennai, Tamil Nadu, India

**Keywords:** Fibro-osseous lesions, juvenile active ossifying fibroma, aggressive ossifying fibroma.

## Abstract

During osteogenesis, mesenchymal tissues function to form fibrous matrix which changes into bone by ossification. In rare instances, fibrous matrix persists in which foci of immature bone is evident resulting in progressive enlargement. Such conditions are commonly benign in nature but few are anatomically benign and clinically destructive. Though recurrence and residual defects following surgical treatment are the challenging complications, fatal consequences are infrequent. We report a juvenile case of ossifying fibroma with an aim to highlight its clinical course and salient criteria to differentiate this entity from the common variants.

## INTRODUCTION

Juvenile ossifying fibroma was first included in the second edition of the WHO classification of odontogenic tumors to describe jaw lesions of children under the age of 15 years. Literature mentions this lesion as aggressive ossifying fibroma and active ossifying fibroma. This fibro-osseous type of neoplasm is nonencapsulated but demarcated well from the surrounding bones, exhibiting rapid enlargement. Thinning and expansion of adjacent cortical bones, eroding the bone to invade the adjacent tissue spaces are the result of its aggressive and active course. Though predominantly occurs in children, 20% of reported cases were above 15 years. JOF is composed of fibrocellular tissues, mineralized materials and small foci of giant cells.^2^ Conservative surgery results in 30 to 50% of recurrence.^3^

## CASE REPORT

A male child aged 4 years was brought to the hospital with a complaint of swelling on the upper anterior teeth region since 15 months. Upon analyzing the history, the father in-formed that, the right upper central incisor was not erupted till the age of 2 and the child was taken to the dental clinic. Dental records stated that opperculectomy was done in 51 region following which the tooth erupted. 6 months later the patient developed a small swelling in the gum region in relation to 51. Swelling was gradually increasing in size with the displacement of incisor teeth and associated with pain.

**Figs 1A to C:**
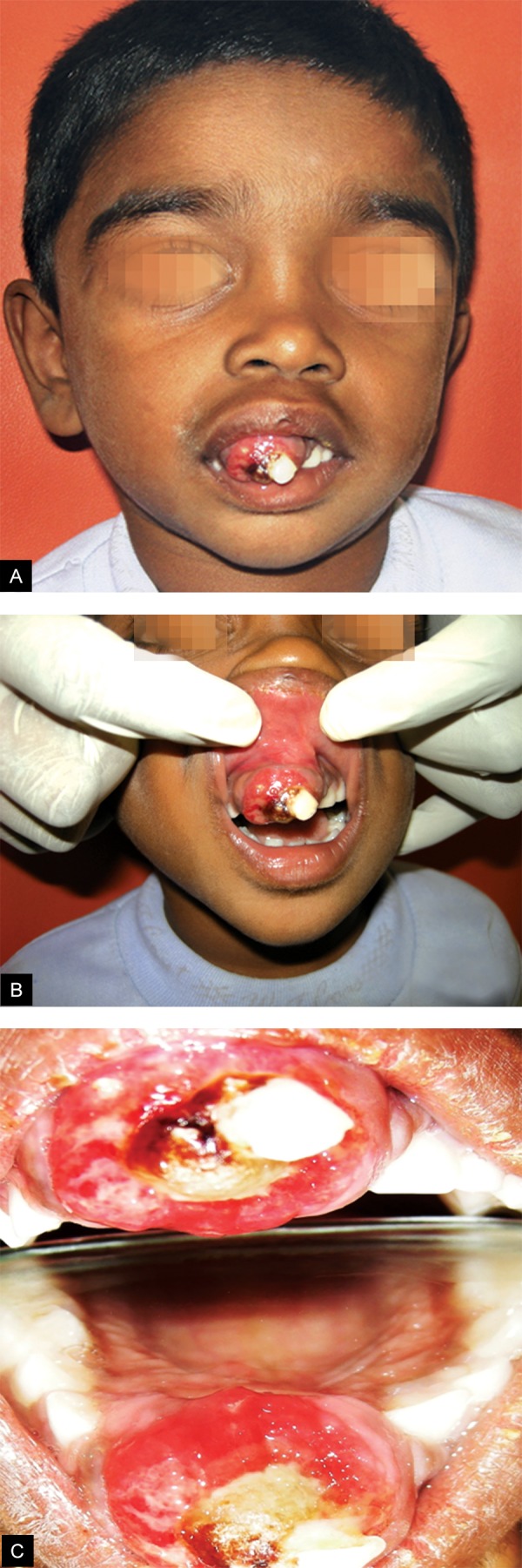
Clinical appearance of the lesion

On examination, a single well-defined sessile growth was evident on the anterior maxillary region encircling the crown of right upper central incisor (Figs 1B and C). The size was approximately 4 × 3 cm anteroposteriorly and mediolaterally protruding between the lips (Fig. 1A). Mouth closure was incomplete. Mucosa overlying the growth was erythematous. Palpatory findings include bony hard consistency, fixation to the deep structures, tenderness and bleeding. 51 and 61 were not mobile and no response was elicited on using electronic pulp vitality tester. No evidence of paresthesia on lip and para-oral structures.

Above features made us to think, bony sarcoma and odontogenic tumor as possible differential diagnosis.

### Radiographic Interpretation

**Fig. 2:**
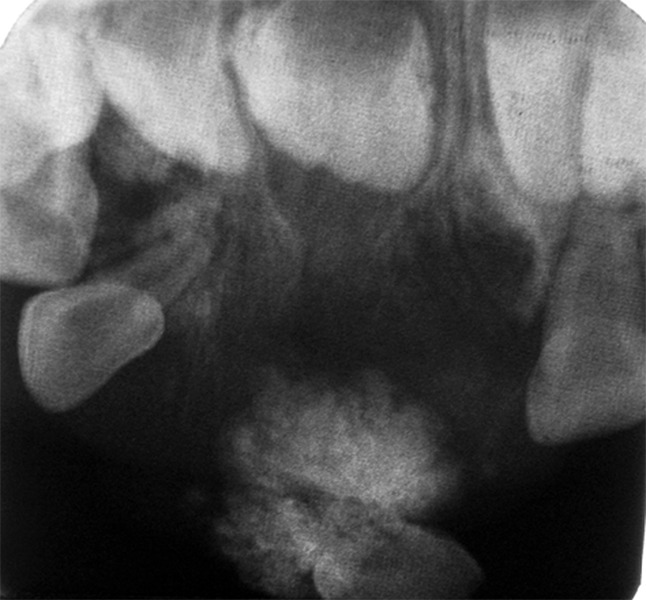
Occlusal radiograph showing mixed type of lesion

Maxillary occlusal view in relation to 51 and 61 region showed well-defined radiopaque mass with numerous specks producing ground - glass appearance between widely displaced 51 and 61. The opaque mass was surrounded by unilocular radiolucency. No evidence of root resorption in relation to 51 and 61. Tooth buds of permanent central incisors were found above the radiopaque mass (Fig. 2).

**Fig. 3:**
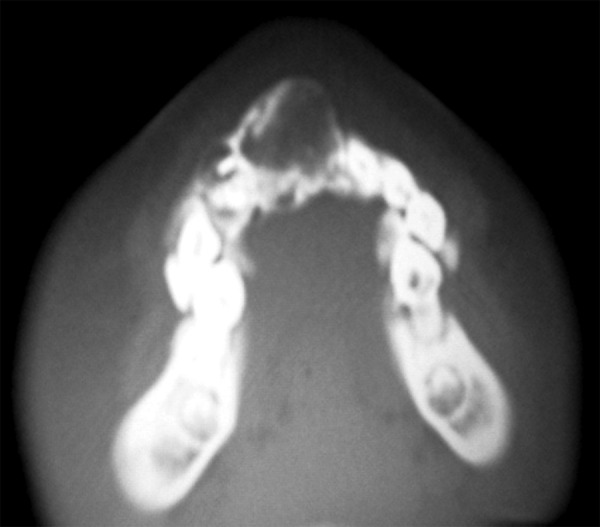
CT maxilla showing cross-sectional view of the lesion

CT maxillary view demonstrated expanded cortex in the anterior alveolar region with ill-defined mixed mass surrounded by areas of corrugated margins (Fig. 3).

**Fig. 4:**
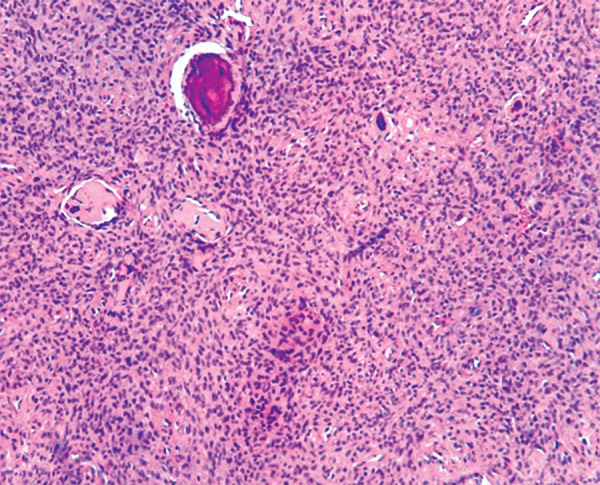
Section shows cellular stroma with basophilic calcification

**Fig. 5:**
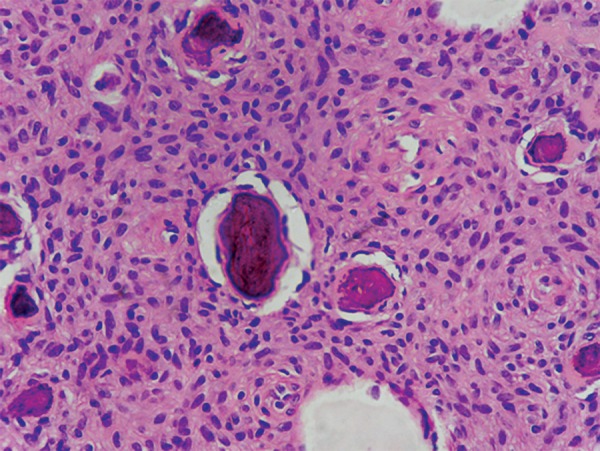
Section shows trabeculae of bone with osteocytes

Microscopic analysis of incisional biopsy specimen demonstrated nonencapsulated rich cellular connective tissue stroma composed of proliferative plumps of fibroblasts along with collagen fibers (Fig. 4). Round to ovoid areas of osteoid and slender trabeculae of woven bone were interspersed in the connective tissue stroma (Fig. 5). Few giant cells were present. No evidence of mitotic figures.

Clinical, radiographic and histopathological corelation suggested the final diagnosis of juvenile ossifying fibroma.

## DISCUSSION

Juvenile ossifying fibroma (ICD-O-code 9262100) was titled by Johnson et al in 1952.^4^ Features to be considered to diagnose JOF include the following:^5-7^

1. Childhood or early adolescent onset of lesion with the history of rapid increase in size and palpatory pain.
2. Maxillary involvement.
3. Radiographic size of the lesion should be greater than 5 cm in diameter.
4. On computed tomography perforation of the expanded cortex is characteristic.
5. Recurrence following treatment.

Nature of the lesion can not be predicted histopatho-logically and often ossifying fibroma is the diagnosis.

Etiology of JOF seems to be De Nova without any apparent causative factor. Two distinct variants of it are psammomatoid juvenile ossifying fibroma (PsJOF) and Trabecular juvenile ossifying fibroma (TrJOF). Majority of PsJOF occur in orbital bone and paranasal sinuses, where as TrJOF primarily affects maxilla followed by mandible.^8,9^ Apart from the anatomical site variation differences between the two types are enumerated in Table 1.

The age and clinical findings of our patients are favorable towards jaw sarcomas. Osteosarcoms are reported in wide range of age group from young children to 3rd and 4th decade of life with high prediction for males.^10^ Trauma is a definite preceding factor and malignant transformation in the rapidly proliferative reparative tissue is the etiopathology. Radiographic findings of mixed radiopaque and radiolucent type of lesions and surgical trauma in our patient’s dental record further supported the clinical diagnosis. Chondrosar-coma also has the similar features with equal frequency in maxilla and mandible.

As the growth involved both labial and palatal aspects of gingiva with bony hard consistency, odontogenic tumor was also considered. Odontogenic tumors (OT) are comparatively rare and comprise about 1% of all jaw tumors.^11^ The study of Buchner et al ^12^ states that the peripheral odontogenic fibroma is the most common type of OT. Clinical manifestations approximate to our patient but age and site are contradictory. Reported mean age was 32.3 years and mandible is the most common site.

Histologically JOF is composed of cell - rich fibroblastic stroma with osteoid matrix incorporating plump eosinophilic osteoblastic cells. Elaboration of mineralized products by the lesion leads to progressive calcification of the osteoid and immature woven bone formation. Focal collections of multinucleated giant cells are common. Collagen is not a usual finding but its presence favor for older lesions. Cystic transformation in tumor mass may sometimes occur and is the consequence of rapid tumor growth in young patients.

On conventional radiograph characteristically the lesion appears as well-defined, radiolucent mass with variable amount of radiopacities. The active zones of growth presents initially as radiolucent locules which become radiopaque later.^12^ Displacement and root resorption of teeth are common at lesion site.

**Table 1:** **Table 1:** Comparison between two pathological forms of JOF^14^

*Sl. No*		*Features*		*PsJOF*		*TrJOF*	
1.		Age		3 months to 72 years with an average range of 10 to 25 years		2 to 33 years with an average range of 8.5 to 12 years	
2.		Sex		M > F 1.1:1		M > F 1.3:1	
3.		Site		Sinonasal > orbit > calvarium > maxilla > mandible		Maxilla > mandible > sinonasal	
4.		Signs and symptoms		Direct intracranial extension results in encephalitis and meningitis		Facial asymmetry, pain and paresthesia. Rarely nasal obstruction epistaxsis and eye displacement	
5.		Capsule		Unencapsulated		Unencapsulated	
6.		Development of aneurysmal bone cyst		Common		Less common	
7.		Radiographic finding		Mixed radiopaque and radiolucent lesions with cystic spaces and incomplete bone shells		Mixed radiodense and radiolucent lesions with expansion and destruction of surrounding bone.	
8.		Histopathological findings		Concentric calcification impart a small, uniform spherical masses of osteoid resembling psammoma bodies dispersed in fibroblastic stroma		Eccentric calcification producing woven bone trabeculae in a cell rich fibroblastic stroma	

Computed tomographic finding of JOF include well defined expansive lesion with variable amount of calcification.

Mixed radiopaque and radiolucent type of lesion that microscopically reveals fibrous stroma with woven bone is unique for fibro-osseous group of lesions and JOF being a member of this group it should be distinguished from the rest of similar lesions. Table 2 summarize the differences.

Periosteal elevation with subperiosteal spread leads to local invasion and indistinct separation between tumor and adjacent tissues make the prognosis uncertain and necessitate more aggressive surgical procedures such as *en block* resection.^13^

In our patient the duration and size of the lesion dictates the slow growing nature of the lesion. Slow growth is the uncommon features of this rare lesion and reported literature are also few^14^

## TREATMENT

**Fig. 6:**
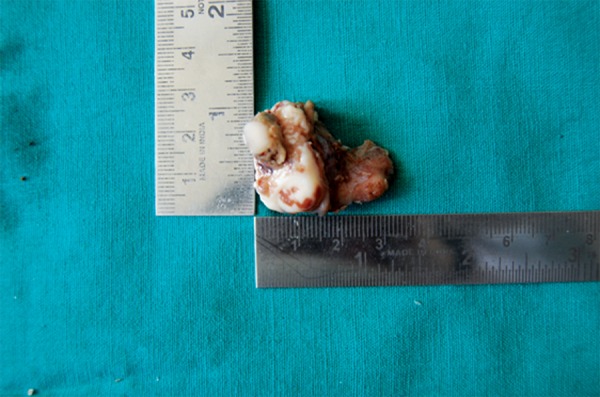
Shows excised surgical specimen

When the treatment plan was decided, the well-defined nature of tumor with no evidence of bony perforation and slow growing tendency were in favor for opting conservative mode of surgery than radical resection. Complete excision of tumor mass was carried out along with teeth followed by curettage of tumor cavity (Fig. 6).

The patient is under observation for the past one year with periodic recall visits and conventional radiographic analysis of the lesion site to verify the resolution.

**Table 2:** **Table 2:** An overview of fibroossious lesions^15^

*S.No*		*Features*		*Fibrous dysplasia (monostotic type)*		*Cemento-osseous dysplasia (COD)*		*Ossifying fibroma*		*TrJOF*	
1.		Etiopathogenesis		Aberrant activity of bone forming mesenchymal tissues due to unrecognized factor		Mid chronic trauma or traumatic occlusion		Unknown		Unknown	
2.		Sex predilection		F = M		F > M		F > M		M>F	
3.		Age		Children and young adults First and second decade of life		Always occur in patients above 20 years age		Young adults with average age of 36 years		Children younger than 15 years	
4.		Clinical features		Painless progressive example lesion		Asymptomatic and only radiographic finding		Slow growing asymptomatic growth that displacement of teeth		Variable rate of growth with pain on palpation and displacement of teeth	
5.		Site		Both jaws get affected. If maxilla is involved it extends its maxillary sinus zygomatic process and floor of mouth		Mandible > maxilla		Mandible > maxilla		Maxilla > mandible	
6.		Radiographic features Mixed radiolucent and radiolucent		Mixed radiolucent and radioh radiolucent lesion giving ground glass or “Puede” “orange” appearance		Mixed type of radiopaque and icent by beginning of calcification and mixed type of appearance and mature stage of radioactive		Initial stage is radiolucent followed radiolucent areas with flecked opacities final radiopaque stage		Early radiolucent intermediate lesion	
7.		Histopathological findings		Fibrous stroma containing proliferating fibroblasts and intercellular collagen fibers and woven bone scattered through out the lesion giving C-shaped pattern		Irrespective of different types, fibroblastic proliferation, woven bone and cementum like materials are the usual components.		Fibrous tissue stroma containing admixtures of bone and cementum like material.		Fibroblastic stroma with osteoid matrix and woven trabecular bone. Focal areas of giant cell are also present.	
8.		Treatment and Recurrent rate		Surgical contouring of the lesion. Treatment should be delayed until cessation of growth spurts. Sarcomatous changes may very rarely occur.		No treatment is required. Rarely simple bone cyst may develop which require surgical management.		Depends on the size of lesion. Enucleation or surgical resection is the common, mode recurrence is rare.		Surgical resection with careful follow-up. Recurrence rate is high.	
